# Supplementary material for: Role of direct and indirect social and spatial ties in the diffusion of HIV and HCV among people who inject drugs: a cross-sectional community-based network analysis in New Delhi, India
Source: eLife. 2021 Aug 3;10:e69174. doi: 10.7554/eLife.69174 (PMC8370773; doi:10.7554/eLife.69174)
Supplement: Supplementary file 1. [file elife-69174-supp1.docx]

| Number of Indexes | 10 |
| --- | --- |
| Median Age (mean) | 38 (38) |
| Proportion Male | 100% (10) |
| Men Who Have Sex with Men | 30% (3) |
| Highest Level of Education |  |
| *No Schooling* | 20% (2) |
| *Primary School (Grades 1 – 5)* | 40% (4) |
| *Secondary School (Grades 6 – 10) or above* | 40% (4) |
| Employed | 90% (9) |
| Ever Tested for HIV | 100% (10) |
| Ever Tested for HCV | 30% (3) |
| Mean Injection Partners in Prior Mo. | 5.7 |
| Median Injections in Prior 6 Mo. (IQR) | 360  (180 – 540) |
| Ever Shared Syringes | 40% (4) |
| Shared Syringes in Prior 6 Mo. | 10% (1) |
| Injected Heroin in Prior 6 Mo. | 50% (5) |
| Injected Buprenorphine in Prior 6 Mo. | 80% (8) |
| Injected Other Drugs in Prior 6 Mo. | 0% (0) |

## 
