# Supplementary material for: Role of direct and indirect social and spatial ties in the diffusion of HIV and HCV among people who inject drugs: a cross-sectional community-based network analysis in New Delhi, India
Source: eLife. 2021 Aug 3;10:e69174. doi: 10.7554/eLife.69174 (PMC8370773; doi:10.7554/eLife.69174)
Supplement: Reporting standard 2. [file elife-69174-repstand2.pdf]

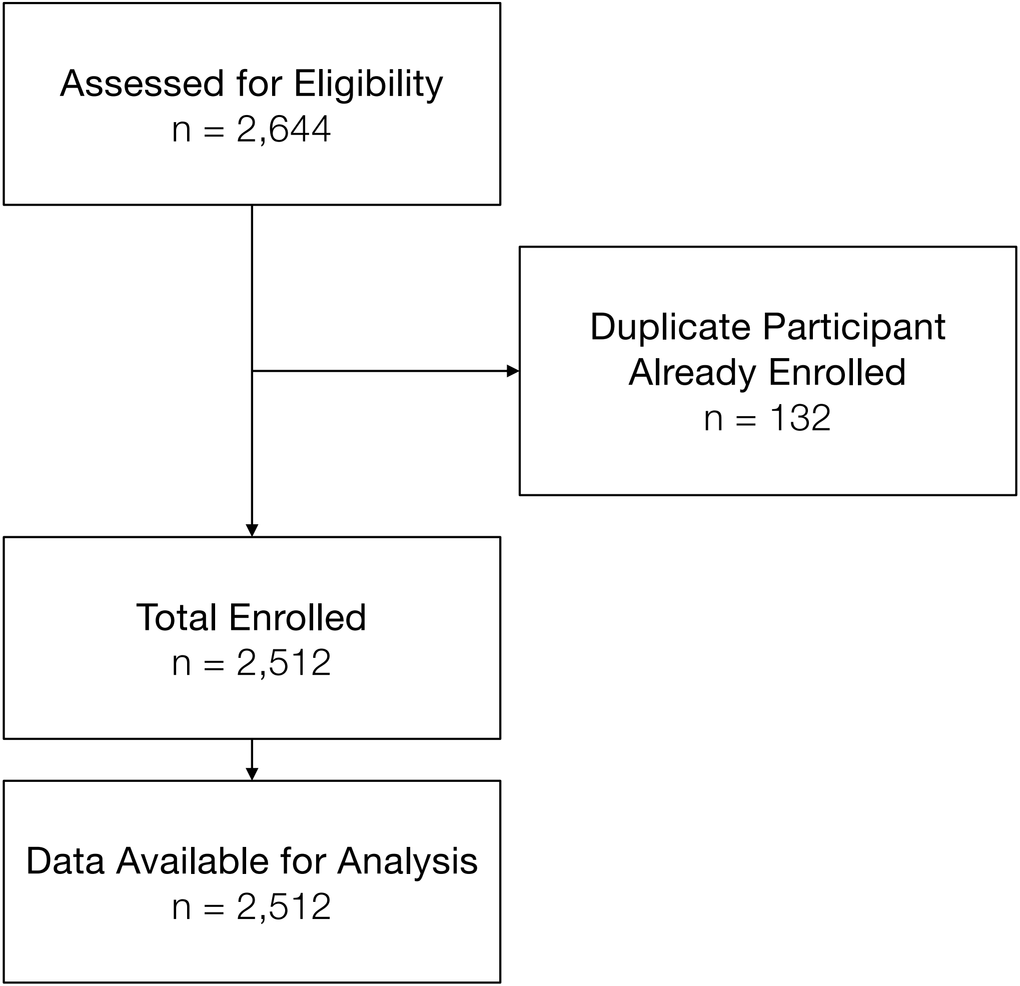
Assessed for Eligibility
n = 2,644
Duplicate Participant
Already Enrolled
n = 132
Total Enrolled
n = 2,512
Data Available for Analysis
n = 2,512
